# Ethical issues and practical barriers in internet-based suicide prevention research: a review and investigator survey

**DOI:** 10.1186/s12910-020-00479-1

**Published:** 2020-05-13

**Authors:** Eleanor Bailey, Charlotte Mühlmann, Simon Rice, Maja Nedeljkovic, Mario Alvarez-Jimenez, Lasse Sander, Alison L. Calear, Philip J. Batterham, Jo Robinson

**Affiliations:** 1 Orygen, Locked Bag 10, 35 Poplar Road, Parkville, VIC 3052 Australia; 2 Centre for Youth Mental Health, University of Melbourne, 35 Poplar Road, Parkville, VIC 3052 Australia; 3 Centre for Mental Health, Swinburne University of Technology, John St, Hawthorn, VIC 3122 Australia; 4 Danish Research Institute for Suicide Prevention, Mental Health Centre Copenhagen, Gentofte Hospitalsvej 15, 4. Sal, 2900 Hellerup, Denmark; 5 University of Copenhagen, Nørregade 10, 1165 København, Denmark; 6 Department of Rehabilitation Psychology and Psychotherapy, Albert-Ludwigs-University Freiburg, Engelbergerstr. 41, D-79085 Freiburg, Germany; 7 Centre for Mental Health Research, Research School of Population Health, The Australian National University, Canberra, ACT 2061 Australia

**Keywords:** Suicide, Research, Ethics, Internet

## Abstract

**Background:**

People who are at elevated risk of suicide stand to benefit from internet-based interventions; however, research in this area is likely impacted by a range of ethical and practical challenges. The aim of this study was to examine the ethical issues and practical barriers associated with clinical studies of internet-based interventions for suicide prevention.

**Method:**

This was a mixed-methods study involving two phases. First, a systematic search was conducted to identify studies evaluating internet-based interventions for people at risk of suicide, and information pertaining to safety protocols and exclusion criteria was extracted. Second, investigators on the included studies were invited to complete an online survey comprising open-ended and forced-choice responses. Quantitative and qualitative methods were used to analyse the data.

**Results:**

The literature search identified 18 eligible studies, of which three excluded participants based on severity of suicide risk. Half of the 15 suicide researchers who participated in the survey had experienced problems obtaining ethics approval, and none had encountered adverse events attributed to their intervention. Survey respondents noted the difficulty of managing risk in online environments and the limitations associated with implementing safety protocols, although some also reported increased confidence resulting from the ethical review process. Respondents recommended researchers pursue a collaborative relationship with their research ethics committees.

**Conclusion:**

There is a balance to be achieved between the need to minimise the risk of adverse events whilst also ensuring interventions are being validated on populations who may be most likely to use and benefit from them (i.e., those who prefer anonymity). Further research is required to obtain the views of research ethics committees and research participants on these issues. Dialogue between researchers and ethics committees is necessary to address the need to ensure safety while also advancing the timely development of effective interventions in this critical area.

## Background

Suicide accounts for close to 1 million deaths worldwide annually, with a crude global rate of 10.6 suicide deaths per 100,000 population. Moreover, for every death by suicide there are up to 30 non-fatal suicide attempts [1, 2]. Suicidal thoughts and behaviours are associated with poor mental and physical health outcomes, including increased risk of suicide, for the individual as well as their friends, family and community [3–5]. Despite the far-reaching negative consequences of suicide and suicidal behaviour, evidence regarding what works in detecting and treating suicidal thoughts and behaviours is still emerging and there remains a need for more high-quality studies in suicide prevention [6–9]. The lack of research in this area has been attributed to several factors, including methodological and practical challenges together with ethical issues [8, 10].

The ethical and practical challenges associated with suicide research are well-documented, with vulnerability of the population, the potential for adverse events, competency of participants to consent, and researcher liability among some of the key issues [11, 12]. Studies that have consulted with research ethics committee members and researchers have therefore unsurprisingly found that research ethics committees, when reviewing applications to conduct research, are particularly concerned with potential risk of harm to participants posed by the research procedures or intervention (including their potential to induce distress or suicidal ideation) and researchers’ ability to respond in the case of distress or risk [13–15]. In order to navigate the ethical and practical issues, and anticipate or address the specific concerns of research ethics committees, researchers in suicide prevention often implement strategies such as excluding participants who are deemed to be at particularly high risk or employing strict safety protocols that may deter some people from participating (e.g., due to privacy concerns) [10, 14–16]. Participants at elevated risk for suicide are also commonly excluded from intervention studies targeting other mental health conditions, such as depression [17, 18]. This effectively means that those who may be in need of the most support are excluded from participating in potentially helpful research, and results in relatively little empirical evidence regarding which interventions are feasible, safe and effective in this population.

Over the past decade, use of the internet to treat mental health problems, including suicidal ideation and behaviour, has become increasingly common [19, 20]. The internet offers many advantages over traditional face-to-face therapy, including wide reach, cost-effectiveness, accessibility and ability to combat stigma-related issues [21–23], with social-media-based interventions also having the potential to reduce isolation and increase belongingness [24, 25]. Internet-based interventions, including smartphone applications, may also have the unique ability to enable real-time monitoring and detection of suicide risk [26]. Despite this, the limited face-to-face contact and protracted availability afforded by the internet means that internet-based interventions are impacted by a range of ethical and practical challenges. The ethical and practical challenges associated with online mental health service delivery to individuals at high risk of suicide have been well studied [27, 28], and management of suicide risk has been embedded into standard ethical codes and guidelines for health practitioners and services [29, 30]. Static, self-directed interventions designed to prevent suicide are increasingly being developed and evaluated [31], but the relative lack of therapist involvement likely gives rise to particular ethical and practical issues.

Studies of such issues in internet-based intervention research more generally have identified confidentiality, data storage and risk management as key challenges [32]; it is likely that these may be amplified when participants are at elevated risk for suicide, particularly where monitoring or detection of risk is a function of the intervention. For example, safety protocols used in face-to-face intervention studies may have limited applicability in environments where researcher contact with participants is limited. To date, however, no research has specifically examined what safety procedures are used in clinical studies of internet-based interventions for people at risk of suicide. While previous studies have sought the views of suicide researchers regarding dealing with research ethics committee concerns [11, 12], no investigation of the ethical issues and practical barriers encountered specifically in internet-based suicide intervention research has been conducted.

The aims of this study were twofold. First, we aimed to describe the safety measures, including inclusion and exclusion criteria, used in published and ongoing studies of internet-based interventions for people who experience suicidal ideation. Second, we aimed to survey suicide researchers regarding their views and experiences in undertaking suicide intervention-based research, particularly with respect to ethical and practical issues.

## Method

### Study design

This was a mixed-methods study involving two phases. In Phase One, studies of internet-based interventions for suicide prevention were identified via a systematic literature search and their reported safety measures were examined. In Phase Two, lead and senior authors of all studies identified during Phase One were invited to participate in an online survey. Following Andriessen and colleagues [14, 15], researchers who participated in the survey will be referred to hereafter as “respondents”; “participants” will refer to the participants of their studies.

### Procedure

#### Phase 1

Medline, PsycINFO and Embase were searched in February 2018 using the following keywords: suicid* AND (app OR application OR internet* OR net OR online OR web* OR e-health OR m-health OR mobile* OR smartphone) AND (intervention* OR program* OR therap* OR treatment*); results were limited to peer-reviewed records published from the year 2000 onwards and available in English. Additionally, abstracts of key suicide prevention conferences (e.g,. International Association of Suicide Prevention World Congress) and internet-based therapy conferences (e.g., International Society for Research on Internet Interventions 9th Scientific Meeting) held in 2017, as well as the World Health Organization International Clinical Trials Registry Platform (ICTRP), were searched to identify research underway. Studies were included if they: 1) tested an internet-based intervention; and 2) recruited participants who experienced suicidal thoughts and/or behaviour. Exclusion criteria were: 1) not an experimental study; 2) participants not recruited based on suicidality; 3) intervention not at least partially internet-based; 4) intervention not targeting suicidal ideation or behaviour. No restriction was placed on study design. All identified studies were hand-searched for information regarding their safety procedures, including inclusion and exclusion criteria related to mental health problems and suicidality. This information was extracted for each included study, as well as other key trial information such as country of origin, participant age and number, trial design (e.g., pre-post, randomised controlled trial), and intervention content.

#### Phase 2

An online survey was developed using Qualtrics survey software. A link to the survey was emailed to the first and last authors of each identified study from the Phase 1 systematic review (or, in the case of registered trials, the principal investigator) in March 2018. Weekly reminder emails (up to four) were sent to those researchers who had not responded to the initial survey invitation. Researchers were also encouraged to inform the research team of any colleagues who were not lead or senior author but who may have had the expertise to respond to the survey.

The survey was purpose-designed for this study based on the authors’ expertise and previous studies in this area, and contained both forced-response and open-ended questions. Questions asked respondents about the general ethical and practical issues associated with internet-based suicide prevention intervention research, previous experience obtaining ethical approval and conducting such research, actions taken to mitigate perceived or actual ethical and practical issues, and the impact of these actions on their research. The survey also asked researchers about anything they would do differently in future studies, as well as their recommendations to other researchers in the field. The survey is available in Additional file 1.

#### Data analysis

For the quantitative data obtained from the literature review and survey, frequencies and percentages were calculated using SPSS. Qualitative data drawn from Phase 2 of the study were analysed using thematic analysis, following the steps set out by Braun and Clarke [33]. The lead author read and re-read the qualitative survey responses to immerse herself in the data. A theoretical semantic approach was used to analyse the data, seeking the answers to four broad questions: 1) What are the ethical and practical issues associated with this research area?; 2) What steps have respondents taken to address these issues?; 3) What impact did this have on the research?; 4) What general recommendations do respondents have for researchers in this area (including what they would do differently in future)? A systematic process to code the data was undertaken, with roughly 10 % of the data independently coded by a second author to ensure the validity of codes developed. The codes were then grouped into themes that captured their shared meaning; themes were discussed to ensure they accurately reflected the data. Data are reported according to the Consensual Qualitative Research Method [34], using the labels “few” (rare support; endorsed by 10–20% of respondents), “some” (variant support; endorsed by 21–50%); “many” (typical support; endorsed by 51–90%), or “most” (general support; endorsed by 91–100%).

## Results

### Phase 1

In total, 877 peer-reviewed articles were retrieved, of which nine met criteria for inclusion. A further nine studies were identified via searching conference abstracts and trial registries; results of two were published during preparation of this manuscript. In total, 18 studies were identified testing 19 interventions.

Key characteristics of the included studies, including their inclusion and exclusion criteria, are displayed in Table 1.

**Table 1:** Characteristics of included studies

Study	Country	Intervention description	Method/ type of evaluation	Participants	Suicide/mental health inclusion criteria	Suicide/mental health exclusion criteria	Safety procedures
**Published studies**							
Hetrick et al., 2017 [35]	Australia	**Reframe IT:** Self-help intervention based on CBT, administered in the presence of school wellbeing staff member. Participants could contact “moderator” via posting on “message board”. No social networking component.	RCT	Secondary school students recruited via school wellbeing staff. N: 50 Mean age: 14.7	**Suicide:** SI in past four weeks **Mental health:** none	**Suicide:** None **Mental health:** Psychotic symptoms, assessed by CAARMS; Intellectual disability	Participants completed a weekly SI screen. If they indicated current SI, a risk assessment was conducted by the school wellbeing staff member, who followed the school’s safety protocols. The website was moderated once daily on weekdays by a psychologist who followed a safety protocol in case of risk (details not reported). It was clear to participants that the website was not moderated 24/7.
Kennard et al., 2018 [36]	USA	**BRITE:** Smartphone application that prompted participants to rate their emotional distress and provided personalised strategies for emotion regulation and safety planning. Supports inpatient intervention, As Safe as Possible (ASAP)	RCT	Adolescents recruited via psychiatric inpatient units at two academic medical centres. N: 66 Mean age: 15.1	**Suicide:** Presented with recent SI + plan or intent OR recent SA **Mental health:** none	**Suicide:** None **Mental health:** None	Not reported
King et al., 2015 [37]	USA	**eBridge:** Online screening + feedback and optional anonymous online counselling (motivational interviewing).	RCT	College students randomly selected from university database and invited to participate N: 76 Mean age: 22.9	**Suicide:** Screened positive for suicide risk (depression and/or SI on PHQ-9; AND/OR reporting history of SA; AND/OR alcohol abuse on the AUDIT) **Mental health:** none	**Suicide:** None **Mental health:** Currently in mental health treatment	All students were provided with a list of mental health resources and received pop-up messages with information about emergency services if SI or a history of SA was reported. No information regarding safety procedures in online counselling component
Melvin et al., 2019 [38]	Australia	**BeyondNow:** Smartphone application for safety planning, used as an adjunct to existing interventions at a mental health service.	Pre-test post-test case series/ open-label single-group trial	Recruited from a tertiary mental health service. N: 36 Mean age: 19.81	**Suicide:** Current or recent SI or behaviour **Mental health:** current engagement with a tertiary mental health service	**Suicide:** None **Mental health:** Psychosis; Intellectual disability	Not reported
McManama O’Brien et al., 2017 [39]	USA	**Crisis Care:** Smartphone application that gives immediate access to a set of coping skills identified as being helpful during a suicidal crisis, and enables user to access help (i.e., call an adult) immediately if necessary.	Pilot test of prototype – reviewed app and provided feedback	Adolescent/ parent dyads recruited recruited from an outpatient psychiatry department. N: 20 Adolescent mean age: 15.7	**Suicide:** Not reported **Mental health:** not reported	**Suicide:** Not reported **Mental health:** Not reported	Not reported
Pauwels et al., 2017 [40]	The Netherlands	**BackUp:** Smartphone application for safety planning, designed as an unguided self-help tool. Link to suicide hotline is permanently visible.	Pre-test post-test pilot study	Adult participants recruited via the Flemish suicide prevention portal and Facebook advertising. N: 21 Mean age: 30	**Suicide:** Some degree of SI (BSS > 1). **Mental health:** none	**Suicide:** None **Mental health:** None	BSS score > 25 triggered phone call by a staff member of the suicide prevention centre to assess suicide risk. All participants received a referral card (at baseline and follow-up) with contact details of mental health care institutions
Robinson et al., 2014 [41]	Australia	**Reframe IT** (as above)	Pre-test post-test pilot study	Secondary school students recruited via school wellbeing staff. N: 21 Mean age: 15.6	**Suicide:** SI in past month **Mental health:** none	**Suicide:** None **Mental health:** Psychotic symptoms, (assessed by CAARMS); Intellectual disability	1) Detailed safety plan completed with each participant after baseline assessment. 2) All assessments and modules were completed at school. 3) Psychological distress and SI were measured weekly, the outcomes of both of these measures were fed back immediately to the school wellbeing staff member.
Tighe et al., 2017 [42]	Australia	**ibobbly:** Culturally-relevant unguided smartphone application for Aboriginal and Torres Strait Islander people, based on acceptance therapies including ACT	Pilot wait-list RCT	Indigenous Australians recruited via posters, Facebook advertising, and through health professionals. N: 61 Mean age: 26.25	**Suicide:** SI in past 2 weeks (SI criteria later removed). **Mental health:** score > 10 on PHQ-9 or > 25 on k10	**Suicide:** “Active intent” as assessed by interview **Mental health:** Diagnosis of schizophrenia or psychotic disorder	In addition to providing helpline and service information, safety checks were conducted via telephone at 3 and 9 weeks. All participants had face-to-face or phone contact with the research officer/ psychologist at baseline assessment, 3 weeks (safety check) and 6-week follow-up. Waitlist group participants also received a further safety check at 9 weeks, and final follow-up assessment at 12 weeks.
van Spijker et al., 2014 [43]	The Netherlands	Unguided self-help intervention based on CBT, but also also makes use of DBT, PST, and MBCT. Participants receive up to six motivating automated e-mails and can ask questions using the FAQ function on the website.	Wait-list RCT	Dutch-speaking adults recruited through online advertisements. N: 236 Mean age: 40.93	**Suicide:** Moderate-severe SI (1–26 on the BSS) **Mental health:** none	**Suicide:** Severe SI (> 26 on the BSS) **Mental health:** Severe depression (> 39 on the BDI)	If participants in either condition exceeded cut-off scores of 26 on the BSS and/or 39 on the BDI, a psychologist contacted them by phone and a conducted a risk assessment. If necessary, their GP was contacted. Participants’ GPs were also contacted if a participant could not be reached by phone.
van Spijker et al., 2018 [44]	Australia	**Living With Deadly Thoughts:** unguided self-help intervention based on CBT, adapted from van Spijker 2014 (see above).	RCT	Australian adults recruited via online media forums including websites, social networking websites, and advertising on search engines. N: 418 Mean age: 40.6	**Suicide:** Current SI (single-item) **Mental health:** none	**Suicide:** SA in the past month (single-item) **Mental health:** History of diagnosed psychotic disorder	At each time point suicide risk was assessed using C-SSRS. Scores of 5 on any item alerted the participant to contact the Suicide Call Back Service (SCBS). Not doing this within 2 days triggered a reminder email to the participant, as well as a message to the SCBS asking them to contact the participant (if contact information was available, otherwise no further action was taken). The provision of contact information to the research team or to SCBS was voluntary.
Wilks et al., 2018 [45]	USA	**iDBT-ST:** self-guided internet-delivered DBT skills training intervention. Participants were emailed fillable DBT worksheets and encouraged to engage in skills practice via daily emails and/or text messaging	Pilot RCT	Recruited via online forums accessible throughout the United States N: 59 Mean age: 38.0	**Suicide:** SI in past month (> 1 on Item 1 of the SBQ-R) **Mental health:** 2+ episodes of Heavy Episodic Drinking AND high emotion dysregulation	**Suicide:** None **Mental health:** Diagnosis of Bipolar I or a psychotic disorder; Enrolled in psychotherapy and unwilling to discontinue treatment	Weekly questionnaires assessing suicide risk were emailed to all participants. Participants endorsing a rating indicating high risk or an increase from the previous week were called and assessed for suicide risk. Additionally, the number for the National Suicide Prevention Lifeline was situated next to prompts on suicidality received by all participants.
**Research underway**							
Courtet, 2018 (NCT03410381) [46]	France	**Ecological Momentary Mental Assessment (EMMA):** smartphone application requiring regular self-assessment of mood, risk and associated factors.	Single-group study – participants complete assessments each month for 6 months	Recruited method not reported Target N: 100 Target age: 18+	**Suicide:** Recent suicidal attempt (< 8 days) or SI (≥ 2 on IDSC-30) **Mental health:** none	**Suicide:** None **Mental health:** None	An action plan is designed to support the participants, to help them facing difficult emotions and feelings, and to encourage them to ask for help. They can contact a relative or call the psychiatric emergency service directly by clicking on a button.
De Jaegere et al., 2016 [47]	Belgium	Self-help intervention based on CBT, developed by van Spjiker 2014 (see above)	RCT	Participants recruited via calls to suicide crisis hotline and via newspaper and online advertising. Target N: 260 Target age: 18+	**Suicide:** Current SI (BSS > 0) **Mental health:** none	**Suicide:** None **Mental health:** None	Any participant who scores > 26 on BSSI and / or > 39 on BDI will be contacted by phone by a clinical psychologist. The psychologist will assess suicide risk, and if necessary, will contact the participant’s GP.
Eylem, 2015 (NL4926 (NTR5028)) [48]	The Netherlands & England	Self-help intervention based on CBT, culturally adapted from van Spijker et al., 2014 (see above)	Wait-list RCT	Turkish migrants, recruited via banners on relevant websites and through social media. Target N: 286 Target age: 18+	**Suicide:** Mild to severe SI (> 1 on the BSS) **Mental health:** none	**Suicide:** None **Mental health:** None	Suicidal thoughts will be assessed once in every 2 weeks. If a participant scores above the cut-off (BSS > 29) then the researcher will ring them to conduct a risk assessment. If the participant does not answer their phone, they will be called for 3 working days at different times of the day. In case of no response after 3 days of attempting to call, a standardised e-mail will be sent asking the participant to contact the research team. The research team will also contact the participant’s GP to inform them about the high SI score. GP will be contacted if necessary following any risk assessment
Larsen et al., 2017 [49]	Australia	**RAFT:** Text messages containing links to information and therapeutic content	Single-group pre-test post-test study	Recruitment method not reported. Target N: 50 Target age: 16–64	**Suicide:** Have presented to an emergency department in the previous 7 days for deliberate self-harm or suicidal behaviours **Mental health:** none	**Suicide:** None **Mental health:** Psychotic disorder	Not reported
Nordentoft, 2016 (NCT02877316) [50]	Denmark	**MyPlan:** Smartphone application for safety planning, used in addition to face-to-face mental health treatment in collaboration with clinician.	RCT	Clients of specialised suicide prevention outpatient service. Target N: 546 Target age: All	**Suicide:** Current/recent suicidality (as indicated by being client of service) **Mental health:** none	**Suicide:** None **Mental health:** Severe alcohol or substance abuse disorder (DSM-V)	Not reported
Mühlmann et al., 2017 [51]	Denmark	Self-help intervention based on CBT, adapted from van Spijker et al., 2014 (see above)	Waitlist RCT	Danish adults recruited via the Danish Lifeline, psychiatric hospitals and outpatient clinics. Target N: 438 Target age: 18+	**Suicide:** SI (> 3 on BSS) **Mental health:** none	**Suicide:** None **Mental health:** None	1) Participants only enrolled in the study after they provide their telephone number and the number of a contact person; 2) BSSI administered every second week over the first 6 weeks, scores ≥27, trigger phone call from trial manager. Trial manager phones emergency contact if participant cannot be reached for 3 days. The same procedure will be carried out if a participant stops using the intervention without notifying the trial manager. 3) Trial website lists contact information for psychiatric hospitals and suicide preventive clinics. 4) Participants encouraged to contact The Lifeline or their GP in crisis. If the trial manager is worried that a participant is at imminent risk, they can obtain access to the participant’s personal identifier number, track the person and request an ambulance or the police.
Nuij et al., 2018 [52]	The Netherlands	2 smartphone applications – **BackUp:** safety planning (as above) **Ecological Momentary Assessment:** self-monitoring Both used together as part of participants’ regular treatment.	Single-group cohort study	Recruited via 3 mental health organisations (current outpatient or day-care patients). Target N: 80 Target age: 18+	**Suicide:** experience SI. **Mental health:** Major depressive disorder or dysthymia	**Suicide:** None **Mental health:** Psychotic symptoms	Not reported

*ACT* Acceptance and Commitment Therapy, *AUDIT* Alcohol Use Disorders Identification Test, *BDI* Beck Depression Inventory, *BSS* Beck Scale for SI, *CBT* Cognitive Behavioural Therapy, *CAARMS* Comprehensive Assessment of At Risk Mental States, *DBT* Dialectical Behavioural Therapy, *IDSC-30* Inventory of Depressive Symptomatology, *MBCT* Mindfulness Based Cognitive Therapy, *PHQ-9* Patient Health Questionnaire-9, *PST* Problem Solving Therapy, *SA* suicide attempt, *SBQ-R* Suicide Behavior Questionnaire-Revised, *SI* Suicidal ideation

Six of the identified studies were conducted in Australia (33%) [35, 38, 41, 42, 44, 49], four in the USA (22%) [36, 37, 39, 45], and eight in Europe (44%) [40, 43, 46–48, 50–52]. Eight studies (44%) tested mobile phone applications [36, 38–40, 42, 46, 50, 52], one tested a text-message-based intervention [49], and the remainder tested computer-based interventions [35, 37, 41, 43–45, 47, 48, 51]. Seven of the interventions were based on Cognitive Behavioural Therapy (CBT; 39%) [35, 41, 43, 44, 47, 48, 51], six specifically on crisis management/safety planning (33%) [36, 38–40, 50, 52], and two on self-monitoring using Ecological Momentary Assessment [46, 52]. One intervention focussed on Dialectical Behaviour Therapy (DBT) [45], one on Acceptance and Commitment Therapy (ACT) [42], and one was a brief contact intervention involving text messages providing links to a range of therapeutic strategies [49]. Finally, one intervention involved screening followed by feedback and optional online counselling [37]. None of the studies identified tested a social-networking-based intervention.

Five studies (28%) specifically targeted young people [35–37, 39, 41], and two (11%) targeted culturally-diverse groups (Aboriginal Australians [42] and Turkish migrants [48]). No other high-risk groups (e.g., older adults, gender and sexual minorities) were specifically targeted in the identified studies. Five (28%) and two (11%) studies required participants to be clients of a mental health service [36, 38, 39, 50, 52] or school counsellor [35, 41] respectively, through whom the intervention was administered. Only three studies (17%) had exclusion criteria related to high suicide risk [42–44]. Ten studies (56%) excluded participants based on other mental health problems [35, 38, 41–45, 49, 50, 52], with nine (50%) excluding participants with psychosis-related disorders [35, 38, 41, 42, 44, 45, 49, 52]. One study excluded participants who were currently accessing mental health treatment [37].

Six (33%) of the 18 studies reported no information about their risk management procedures [36, 38, 39, 49, 50, 52]. Of the twelve that did provide information, seven (58%) described active involvement of the research team in risk management procedures [40, 42, 43, 45, 47, 48, 51], and four (33%) described contact with the participants’ emergency contact or general practitioner (GP) [43, 47, 48, 51].

### Phase 2

#### Respondents

As some researchers were authors on more than one study identified in Phase 1, only 30 individual researchers were invited to participate. No additional researchers were suggested to the research team by respondents. Fifteen completed the survey, giving a response rate of 50%. Seven respondents (47%) were from either Australia or New Zealand, six (40%) were from Europe, and two (13%) were from the USA. The median years of experience in mental health and suicide prevention research was 15 and 10 years respectively. Respondents had been involved in a median of three studies that specifically examined internet-based interventions for people at risk of suicide.

#### Quantitative analysis

Responses to the binary forced-choice survey questions are displayed in Table 2. As can be seen, about half of respondents had experienced problems obtaining ethics approval and recruiting participants. Eleven (73%) thought the safety measures implemented negatively impacted the validity of their project/s. No respondents had experienced adverse events or serious adverse events attributed to their intervention. Many (80%) had taken measures to monitor and/or protect the mental health of the research team, however the details of these were unclear from the data collected.

**Table 2:** Forced-response survey questions

	Yes % (n)	No % (n)
Have you experienced any problems obtaining ethical approval?	47 (7)	53 (8)
Have you experienced problems recruiting participants?	53 (8)	47 (7)
Did you feel your sample was/is representative of people who would use the intervention if freely available?	40 (6)	60 (9)
Did you feel the safety measures were adequate?	80 (12)	20 (3)
Did you feel the safety measures affected the validity of the project?	73 (11)	27 (4)
Have you encountered any adverse events or serious adverse events during the course of your research that were attributable to the intervention/s you were testing?	0 (0)	100 (15)
Did you take any steps to monitor/protect the mental health of the research team?	80 (12)	20 (3)

Additionally, of the eight respondents who had been involved in evaluations of face-to-face interventions for people at risk of suicide, all agreed that there were differences in the ethical and practical issues encountered between online and offline intervention research; these differences were expanded on using free-text responses and are included in the qualitative analysis.

#### Qualitative analysis

Qualitative results are reported according to the four key questions: 1) What are the ethical and practical issues associated with this research area?; 2) What steps have respondents taken to address these issues?; 3) What impact did this have on the research?; and 4) What general recommendations do respondents have for researchers in this area?

#### What are the ethical and practical issues associated with this research area?

Five main themes were identified relating to risk and risk management, participant rights, impact on research, and researcher and ethics committee perspectives. These are described below.

##### Adequate management of risk

Many (53%) respondents noted issues related to the adequate assessment and management of risk to participants. In particular, respondents noted the difficulty of assessing, monitoring and responding to risk in online environments, with one stating, *“It is much easier to assess risk and safety face to face, and in [face to face] studies you have the capacity to remain with a person until help arrives and that is not possible in online studies.”* A few (13%) also expressed concerns that participants may under-report their risk or provide false contact information, making the assessment and management of risk more difficult.

##### Risk of harm to participants

Some (27%) respondents were concerned about potential risk of harm to participants as a result of research participation or exposure to the intervention. A few (13%) were also concerned that a participant may die by suicide during study participation, with one specifically referring to the ethical issues associated with death by suicide whilst allocated to a wait-list control group.

##### Conflicting views between researchers and ethics committees

Some (40%) respondents mentioned inconsistencies between the views of research ethics committees and the views of researchers, due to the perception that research ethics committees may lack knowledge about the safety of conducting research with individuals who experience suicidal ideation and/or being too risk-averse. One respondent stated, “*ethics committees do not understand that in many cases … people are accessing suicide related websites anyway, and in fact what is being provided in the research is … over and above what they usually get in terms of information and support*.”

##### Protection of participant privacy

A few respondents (20%) raised issues related to protecting participants’ privacy: one was concerned about safe storage of data, one about breaching confidentiality when managing suicide risk, and one about the possibility that participants may publicly share sensitive information about other participants.

##### Difficult to achieve target sample size

Many (67%) respondents discussed difficulties they had experienced in reaching their target sample size by failing to recruit the desired number of participants at baseline and/or losing a high proportion of participants to follow-up. Some (27%) indicated that recruiting via third parties (e.g., mental health services or school counsellors), was problematic in terms of recruiting the required number of participants because third parties either unintentionally or deliberately failed to refer participants to the research.

#### What steps have respondents taken to address these issues?

Four main themes were identified, relating to risk management protocols, informing participants, expertise of the research team, and adapting the study design. These are described below.

##### Implementing risk management protocols

Many (53%) respondents said that they had implemented a risk management protocol (also referred to as a safety protocol or procedure) in order to mitigate against many of the ethical and practical issues described above. Risk management protocols typically required participants to provide contact information including a phone number, and usually either provide the contact information of a GP or other mental health professional; together these requirements restricted the ability of participants to take part anonymously.

##### Ensuring participants were informed

Some respondents (27%) reported the importance of having informed consent procedures that clearly communicated to participants the steps taken to manage risk and/or the limits of the internet intervention to respond in a crisis.

##### Collaborating and seeking expertise

A number of respondents discussed taking steps around the theme of seeking expertise. For example, a few (13%) emphasised the importance of including individuals with clinical training on the research team. Others (13%) mentioned they had collaborated with key stakeholders, including clinical staff at recruitment sites or members of the target population, in an attempt to ensure both recruitment and risk management procedures were feasible and likely to succeed. Finally, a few (13%) respondents had open discussions with their reviewing research ethics committee at the outset of the project and/or throughout the review process. For example, one said, *“we had discussions with the ethics committee to get a sense of what we might be able to do and designed our protocol accordingly … [which] meant we did not have major problems down the track.”* Indeed, several respondents implied that they anticipated ethical issues in the planning of their studies and took steps to proactively address these accordingly, ensuring a smoother process for the ethics approval process.

##### Adapting study design to facilitate recruitment

In addressing difficulties with recruitment, some (47%) respondents reported that they altered their recruitment strategy and/or timelines. Steps taken included broadening inclusion criteria, extending the recruitment period, advertising more widely, offering incentives for participation, and allowing participant interviews to be conducted via phone rather than face-to-face.

#### What impact did addressing the ethical and practical issues have on the research?

Two main themes were identified relating to generalisability of the sample and impact of safety procedures. These, together with other negative and positive impacts raised by respondents, are described below.

##### Limited generalisability of the sample

Many (67%) respondents reported that the steps taken to address ethical and practical issues had an impact on the representativeness and generalisability of the sample; some (33%) believed that the lack of anonymity required to ensure effective safety procedures precluded many potential participants, who would usually prefer to remain anonymous, from participating. For example, one respondent said, “*… a certain type of people who think anonymity is very important and for whom an online, anonymous intervention would be very good, refused participation*”. The inclusion criteria applied by third party referrers was also believed to have a negative impact by a few (13%) respondents, with one saying *“while our inclusion criteria … were broad, those who were referring young people to the study were imposing their own inclusion criteria in practice by only referring those they thought could cope with being part of research.”.* Additionally, although most respondents had not excluded participants based on severity of suicidal ideation, those who did suggested this limited the generalisability of the results to lower-risk populations. For example, one respondent said, “*Excluding individuals who are at extremely heightened risk of suicide clearly has limitations in terms of knowing how the intervention will work for this group.”* Another said, *“we have often had situations where we have had to exclude individuals who are ‘too suicidal’ from participating, which means that we will never really know whether these interventions have benefits (or cause harm) to the very individuals whom they are targeting.”*

##### Risk management protocols are an intervention

Some (47%) respondents noted that risk management protocols could be considered an intervention in themselves, making it difficult to truly ascertain effects of the intervention. One respondent said, *“The safety procedures … in control groups had positive effects, decreasing the differences with the positive effects of the experimental groups, rendering it more difficult to obtain true effects of the intervention.”* Another respondent commented on the difficulty associated with measuring, and thus controlling for, implementation of risk management protocols.

##### Other negative impacts

Delays or additional costs to the study, and additional burden on research staff, were reported by individual respondents as other negative impacts of addressing the ethical and practical issues. Additionally, a few respondents (20%) reported concerns that participants would under-report their risk to avoid activating safety protocols, and that activation of the safety protocols was distressing for some participants.

##### Steps taken can have positive impacts

A few respondents (20%) reported positive impacts of the steps taken to address ethical and practical issues. These included allowing researchers to proceed with their research, having an opportunity to educate the research ethics committee, and increased confidence of the research team (e.g., with regard to managing participant risk). One said, *“[the changes have] given us confidence and allowed us to test interventions that we might not otherwise have been able to test.”*

#### What general recommendations do respondents have for researchers in this area?

Two main themes were identified relating to inclusion criteria and seeking expertise. These, together with additional recommendations identified, are described below.

##### Inclusion criteria should be as broad as possible

Some (47%) respondents suggested that ideally inclusion criteria used in future studies should be as broad as possible and include participants at the severe end of the suicidality spectrum; however, some (27%) also suggested that inclusion criteria, particularly related to risk, would ultimately depend on many factors including the type of intervention and level of contact with participants. A few (13%) thought participants should be able to participate anonymously. One respondent discussed the difficulty between allowing for anonymous participation whilst also managing risk appropriately: “*ideally I would run a trial that would allow anonymous participation with little restriction on severity levels. However, complete anonymity would severely limit the potential for a safety protocol.”*

##### Seek expertise

Some (47%) respondents spoke about the importance of collaborating with others, including key stakeholders, referrers, and research ethics committees, in the design and delivery of internet-based suicide prevention intervention research, with one saying, “*It could be worth talking to your ethics committee about your rough research plans early on to avoid problems or having to change your design significantly later”.* A few (13%) respondents highlighted the necessary expertise of the research team, in terms of clinical training and experience in data management.

##### Other recommendations

Other recommendations were mentioned by a few (20%) respondents, including consideration of the real-world implementation of the intervention, monitoring the wellbeing of research assistants, and ensuring participants truly stand to benefit from study participation. One respondent stated, *“Always consider the suicidal participant and whether your research will actually help them or you. Will the intervention make the person more distressed, stressed or suicidal? Does it need a waitlist or controlled design for ‘better science’? If you knew a participant would suicide during your study, is there anything you would change in the design if given the chance? Always treat participants as clients who deserve respect and care rather than research participants.”*

## Discussion

This is the first study to examine ethical and practical issues associated with internet-based intervention research for people who experience suicidal thoughts or behaviours. The findings are discussed here in order of their perceived importance. Results indicate that trials of internet-based interventions for suicidality are characterised by many of the same challenges associated with suicide prevention research, and indeed human research more generally. These include: concerns about potential harm to participants; risk management; recruitment difficulties, particularly where third party referrers are used; conflicting views of research ethics committee members and researchers; and the impact of inclusion and exclusion criteria on sample generalisability [8, 11]. Unique issues were also identified; for example, the assessment and management of risk, appear to be more complex in internet-based intervention studies compared to face-to-face studies, particularly where the amount of contact between participants and the research team or a third-party mental health professional is limited. Interestingly, issues relating to safe storage of data or participant competency to consent did not emerge as prevalent themes; this contrasts with the literature regarding online and suicide prevention interventions more generally [14–16, 32].

A key finding of this study relates to the limitations associated with adequately managing participant risk, which typically involves collecting participants’ contact information together with information about other mental health or medical professionals involved in their care. First, safety protocols were perceived to be a form of intervention in themselves, leading to difficulty determining efficacy of the intervention being evaluated. The interventional effect of the comparison group is a common methodological limitation of clinical trials with high risk populations, where true control conditions are not viable due to the ethical implications associated with withholding treatment [53, 54]. Safety protocols were also viewed to preclude anonymous participation and thus reduce the representativeness of the sample above and beyond any pre-defined exclusion criteria. Given that one of the most commonly-cited benefits of internet interventions for mental health problems is their ability to combat stigma-related barriers by allowing anonymous participation [21–23], this finding implies that online suicide preventive interventions are not being tested with the populations who may be most likely to use them; therefore, the external validity of trials may be limited. Moreover, difficulties obtaining target sample sizes, with consequent reductions in power to detect effects, was a common experience for survey respondents and is consistent with the literature regarding challenges in suicide prevention research, and health research more generally [8, 55]. These recruitment challenges may be somewhat mitigated by allowing anonymous participation where possible. Alternative options to complete anonymity, such as conditional anonymity wherein contact details are only accessed in case of emergency, may also be worthy of exploration in future.

Internet-based interventions are a relatively new area in mental health and despite an emerging literature to support their safety and efficacy in populations at elevated risk of suicide there is more work to be done. Considering the shortage of available evidence, together with the reality that people at elevated risk of suicide may be particularly vulnerable, it is unsurprising that research ethics committees, who are mandated to weigh the benefits of research participation against possible risks [56], are concerned about the potential for harm. Indeed, half the respondents in this study had experienced problems obtaining ethics approval, and this proportion would likely be larger had respondents not tended to anticipate the concerns of the research ethics committee in designing their studies. Although the lack of adverse events reported by respondents in this study provide preliminary support for the safety of internet-based intervention studies in suicide prevention, it is acknowledged that the true occurrence of such events may be under-reported [57]. Relatedly, the finding that most studies did not exclude participants based on severity of suicidality must be interpreted in context of qualitative data suggesting that third-party referrers impose their own criteria regarding “readiness” to participate in research. Overall, then, while this study provides emerging support for the safety of internet-based interventions for people at risk of suicide, this should be interpreted with caution as it may not reflect their real-world implementation.

Although a broad range of studies was identified by the literature review, the evidence base is limited in several areas. Internet-based interventions that facilitate social networking between participants, and those developed for populations known to be at specific risk of suicide (such as those with suicidality in the context of psychosis, older adults, and sexual and gender minorities), may be worthy of further exploration. Consistent with recommendations for clinical research more generally [58], respondents emphasised the importance of collaboration and open dialogue with research ethics committees, however there was little mention of involving consumers, including those with lived experience of suicidal behaviour, in the design of studies. Additionally, there were inconsistencies in the level of detail reported in the published literature regarding safety protocols, which is surprising given the emphasis on safety and risk management. Finally, although most respondents had taken steps to monitor the wellbeing of the research team, which is promising given the potential for suicide prevention researchers to experience negative mental health outcomes such as vicarious trauma [59], little could be concluded regarding the nature and quality of such strategies from the data collected. Future research in this area is encouraged to address these gaps in research. Such research would benefit particularly from the inclusion of consumers with lived experience in the design of interventions and trials, and from transparency in the reporting of safety protocols, adverse events, and procedures to monitor and protect the wellbeing of the research team.

### Limitations and future directions

In interpreting the findings of this study, several limitations should be considered. First, the survey respondents represented a range of countries and their responses were based on experiences with different ethics jurisdictions. Although it is likely that there are differences in research ethics committees between countries and institutions, the small sample precluded examination of these differences. Future research should examine whether research ethics committees’ responses to internet-based suicide-related research vary across countries and institutions. Second, respondents were not required to name the specific projects they were referring to; it is therefore possible that multiple respondents provided data based on the same projects. A third limitation, also related to the sample, was the self-selected nature of participation. It cannot be ruled out that suicide researchers with more negative experiences of online interventions, such as those who had encountered serious adverse events, chose not to participate. Alternatively, it is possible that researchers with positive experiences felt no need to participate whereas those with negative experiences participated to vent their frustration. Fourth, the inclusion criteria related to intervention type were broad, and diverse interventions were included. Although not possible in the current study, as the field matures research should aim to explore how ethical and practical barriers vary based on specific intervention factors, such as the extent of contact facilitated between participants and mental health professionals, the research team, or other research participants. Fifth, it was beyond the scope of this project to seek the views of research ethics committees or research participants with lived experience of suicidal thoughts and behaviours; future research should endeavour to do so and explore how these may vary between groups. Finally, it is acknowledged that several of the ethical and practical challenges described in this study are not unique to internet-based suicide prevention research, and often characterise intervention research more broadly. Future research could expand on the differences between research ethics committee feedback across different study designs and disciplines, for example through comparative qualitative analysis.

## Conclusion

This is the first study to characterise the ethical and practical challenges experienced when testing internet-based interventions for suicide prevention. Although it appears research into these interventions can be conducted safely, various factors limit their real-word relevance. There is a balance to be achieved between minimising the risk of encountering adverse events and ensuring internet-based interventions are being validated within the high-risk populations who may be most likely to use or benefit from them. Further research should seek the views of research ethics committees and consumers with lived experience of suicidal thoughts and behaviours in order to better understand the ethical challenges from the perception of these key stakeholder groups. Researchers should endeavour to establish a collaborative relationship with their research ethics committees in order to ensure risk of harm to participants is reduced while also advancing the timely development of effective and relevant interventions in this critical area.

## Supplementary information

**Additional file 1.** Survey.

## Data Availability

Data supporting the results of this article is not publically available as the individual privacy of participants may be compromised.
