# Supplementary material for: Ethical issues and practical barriers in internet-based suicide prevention research: a review and investigator survey
Source: BMC Med Ethics. 2020 May 13;21:37. doi: 10.1186/s12910-020-00479-1 (PMC7222514; doi:10.1186/s12910-020-00479-1)
Supplement: Supplementary file 1 — Additional file 1. Survey. [file 12910_2020_479_MOESM1_ESM.docx]

# Additional Files

**Additional File 1: Survey**

1. For how many years have you worked in *mental-health* research?
2. For how many years have you worked in *suicide prevention* research?
3. How many studies have you been involved with that specifically examine internet-based interventions for people who are at risk of suicide?

**The remainder of the questions in this questionnaire refers to research projects you have conducted with participants who are at risk of suicide.**

1. Have any of these projects focussed on a specific demographic population?

- Yes - Please indicate which population/s:
  1. Young people (i.e. up to the age of 25)
  2. Aboriginal, indigenous or first nations people
  3. Older adults (i.e. 65+ years)
  4. Culturally and linguistically diverse people
  5. People who have been bereaved by suicide
  6. Males only
  7. Females only
  8. Lesbian, Gay, Bisexual, Transgender, Intersex, or Queer/Questioning (LGBTIQ) people
  9. Other (please describe) ____
- No

1. What was your role (or roles) in these projects (select all that apply)?
   - Planned the study
   - Prepared ethics applications
   - Supervised junior members of the study team
   - Collected data from participants face-to-face
   - Collected data from participants over-the-phone
   - Collected data from participants online
   - Analysed/interpreted data
   - Written or disseminated the study results
   - Moderated or monitored the internet-based intervention
   - Other (please describe)_____
2. What, in your view, are the benefits of internet-based interventions for people who are at risk of suicide (please select all that apply)?
   - Availability
   - Accessibility (i.e. by people in remote areas)
   - Anonymity
   - Other (please specify) _____________________
3. What, in your view, is the **biggest benefit** of internet-based interventions for people who are at risk of suicide?
   - Availability
   - Accessibility (i.e. by people in remote areas)
   - Anonymity
   - Other (please specify) _____________________
4. What, in your view, are the limitations of internet-based interventions for people who are at risk of suicide (please select all that apply)?
   - Discourage people from seeking face-to-face help
   - Limited capacity of professionals (i.e. moderators) to respond in a crisis
   - Risk of contagion
   - Risk of people entering into suicide pacts
   - I do not believe there are any limitations of internet-based interventions for people who are at risk of suicide
   - Other (please specify) __________
5. What, in your view, is the **biggest limitation** of internet-based interventions for people who are at risk of suicide?
   - Discourage people from seeking face-to-face help
   - Limited capacity of professionals (i.e. moderators) to respond in a crisis
   - Risk of contagion
   - Risk of people entering into suicide pacts
   - I do not believe there are any limitations of internet-based interventions for people who are at risk of suicide
   - Other (please specify) __________
6. What do you think the biggest barriers are to conducting internet-based intervention research with people who are at risk of suicide?
   - Difficulties with recruitment
   - Difficulties obtaining ethical approval
   - Lack of adequate resources to be able to respond to risk
   - Other (please specify) ___________
7. What ethical problems did you anticipate encountering before starting your project/s? What measures (if any) did you take to address these?
8. Did you personally have any concerns about the ethical nature of your project/s?
   - Yes
     1. What were these?
   - No
9. Did you experience any problems in obtaining ethical approval for the project/s?
   - Yes
     1. What problems did you experience?
     2. What was the ethics committee most concerned about?
     3. How did you address these concerns?
     4. Did this affect the project/s in any way?
   - No
10. Did any ethical issues arise during the project/s that you had not anticipated?
    - Yes
      1. What were these?
      2. How did you address these?
    - No
11. Did you experience any problems recruiting participants to the project/s?
    - Yes
      1. What were these?
      2. How did you address these?
    - No
12. Did any other practical issues arise during the project/s that you had not anticipated, in relation to the nature of the population?
    - Yes
      1. What were these?
      2. How did you address these?
    - No
13. Did you exclude any participants based on their level of suicide risk (e.g. too mild or too severe)?
    - Yes
      1. What were the exclusion criteria?
      2. How and why did you exclude participants on this basis?
    - No
14. Did you exclude any participants based on symptoms or diagnosis of any psychiatric disorder?
    - Yes
      1. Why did you exclude participants on this basis?
    - No
15. Do you feel your sample was/is representative of people who would use these interventions once they are made freely available?
    - Yes
    - No
      1. Why/why not?
16. If you were to conduct this project/s again, would you use the same inclusion/exclusion criteria?
    - Yes
    - No
      1. What would you change?
17. In general, what inclusion and exclusion criteria do you think should be employed by researchers conducting studies of internet-based interventions for people at risk of suicide?
18. What safety measures did you employ for your project/s?
    - Moderation of the intervention (i.e. checking for posts made by participants indicating they might be at risk)
      1. Please describe: ____
    - Risk escalation and management protocols (i.e. how to identify and manage suicide risk in participants)
      1. Please describe: _____
    - Discontinue procedures (i.e. how and when to withdraw participants from the research)
      1. Please describe: _________
    - Collecting emergency contact information
      1. Please describe: ____________
    - Other
      1. Please describe: _______
19. Do you feel that the safety measures you included in your project/s were adequate?
    - Yes
      1. Please explain your answer:
    - No
      1. Please explain your answer
20. If you were to conduct the project/s again, would you make any changes or refinements to these measures?
    - Yes
      1. If yes, please describe what changes you would make:
    - No
21. Do you feel that the safety measures affected the validity of the project/s in any way?
    - Yes
      1. If yes, please explain why you think this is the case:
    - No
      1. If no, please explain why you think this is the case:
22. Have you encountered any adverse events or serious adverse events during the course of your research that were attributable to the intervention/s you were testing?
    - Yes
      1. If possible, please provide details (i.e. what happened and why)
    - No
23. Did you take any steps to monitor or protect the mental health of the researchers involved in the project/s?
    - Yes
      1. What steps did you take?
    - No
24. If you were to conduct the project/s again, is there anything else you would have done differently?
25. Have you conducted research examining the efficacy of face-to-face interventions for people who are at risk of suicide?

- Yes
  1. Have you found are any differences in the ethical or practical issues you faced in studies of online vs offline interventions?
  2. Please describe these differences:
- No

1. Is there any advice you would give to other researchers who want to conduct internet-based intervention research with people who are at risk of suicide?
2. Do you have any other comments?
